# The convergence paradox: a constructivist account of perceived template convergence and originative agency under generative artificial intelligence

**DOI:** 10.3389/fpsyg.2026.1924628

**Published:** 2026-09-16

**Authors:** Qi Cao, Ziran Zhou, Lin Wang, Yutong Liu, Yifan Li

**Affiliations:** 1 Department of East Asian Studies, Graduate School of International Studies, Hanyang University, Seoul, Republic of Korea; 2 Department of Mathematics, Faculty of Science, National University of Singapore, Singapore; 3 School of Humanities, Arts and Education, Shandong Xiehe University, Jinan, China; 4 College of Performing Arts and Sport, Hanyang University, Seoul, Republic of Korea

**Keywords:** algorithmic homogenization, creativity, generative artificial intelligence, human agency, meaningful work, perceived template convergence, personal construct theory, proxy agency

## Abstract

Generative artificial intelligence (AI) has raised a widely shared concern that human cognitive and creative output is drifting toward common templates. The terms used to describe this drift, including algorithmic monoculture, outcome homogenization, and the loss of collective diversity, all characterize systems, populations, or model outputs. Psychology has not supplied a matching construct at the level of the individual, that is, a way to describe how a person perceives such convergence and how that perception reshapes agency. This article introduces and analyzes perceived template convergence (PTC), defined as a person’s appraisal that the construals available to her through generative AI are collapsing toward a shared template. Working from personal construct theory, the agentic strand of social cognitive theory, and research on meaningful work, the analysis argues that PTC is psychologically two-sided. The appraisal that raises a person’s confidence in producing competent output through AI can also weaken her sense of authorship and meaning, steering effort toward imitative production and away from the more demanding work of origination. We call this structure the convergence paradox, set it out as a propositional model with two mediators and two boundary conditions, distinguish PTC from neighboring constructs, and propose a measurement agenda. The contribution recasts algorithmic homogenization as a question about human construing rather than about outputs.

## 1 Introduction

Generative artificial intelligence (AI) has become, in a remarkably short time, a routine instrument of thought. Large language and multimodal models now help people draft documents, design images, write code, and reason through decisions, and for a growing number of users these systems function less as occasional aids than as standing partners in cognitive and creative work (Brynjolfsson et al., 2025; Noy and Zhang, 2023). The shift has revived an old anxiety in a new form. As more people think through the same small set of models, observers worry that the work they produce, and perhaps the habits of mind behind it, is converging on a common pattern. The worry has empirical support. In a controlled writing study, Doshi and Hauser (2024) found that access to generative AI raised the judged creativity of individual stories while making the stories more similar to one another, so that a gain in individual novelty coincided with a loss of collective diversity.

Researchers have referred to this drift by several names. Kleinberg and Raghavan (2021) describe algorithmic monoculture, the condition in which many actors who rely on a shared algorithm produce correlated, and sometimes collectively worse, decisions. Related work speaks of outcome homogenization, in which shared models and data pull the outputs of different people toward one another (Zhang et al., 2025), and of model collapse, in which systems trained on their own prior output lose the richness of their distribution (Shumailov et al., 2024). Each label captures something real. Each, however, names a property of an aggregate: the correlation among decisions, the variety of an output distribution, the behavior of a data-generating process. None of them names a person’s state.

This omission matters because the question that gives the topic its urgency is psychological. Whether algorithmic homogenization erodes human creativity, autonomy, or motivation cannot be settled by describing outputs alone, since the relevant effects lie in how people experience and respond to convergence. Psychology, however, has yet to supply a construct pitched at that level. The constructs that come nearest belong to adjacent problems. Processing fluency concerns the ease of taking in a single stimulus, not the perceived similarity of a set of options (Reber et al., 2004; Schwarz et al., 2021). Algorithm appreciation and algorithm aversion concern willingness to use algorithmic advice, not appraisal of whether that advice is converging (Dietvorst et al., 2015; Logg et al., 2019). The phenomenon that Doshi and Hauser (2024) measure across a body of outputs has no settled counterpart inside the person who produces them.

The present article supplies that counterpart. We define perceived template convergence (PTC) as a person’s appraisal that the construals she can obtain through generative AI are collapsing toward a common template, that is, a sense that the tools available to her, and to everyone like her, tend to return the same kind of answer. PTC is an appraisal rather than a measurement. It may correspond closely to objective homogenization, loosely, or not at all, and the analysis treats the appraisal itself as the variable that carries psychological weight. Casting the construct at this level allows the questions raised by the homogenization literature to be posed to minds rather than aggregates.

The article develops a constructivist account of why this appraisal should be consequential, and why its consequences should pull in two directions at once. Working from Kelly’s (1955) personal construct theory, we treat an AI template as a construal supplied from outside the person, one that can stand in for her own construal. Studying from the agentic strand of social cognitive theory (Bandura, 2001, 2006), we treat reliance on convergent templates as a case of proxy agency, with the efficiencies and the risks that Bandura attributes to it. Working from research on meaningful work (Rosso et al., 2010), we connect convergence to the experience of authorship, which gives work much of its significance. The combination yields the article’s central claim, the convergence paradox: the same appraisal of convergence that raises a person’s confidence in producing competent output through AI can also lower her experienced authorship, so that effort is drawn toward reproducing the prevailing template and away from the costlier work of departing from it.

The remainder of the article proceeds as a conceptual analysis (Attwood, 2022), advancing argument, definition, and a propositional model rather than reporting new data. The next section examines the homogenization literature and locates its missing level of analysis. The two sections that follow define PTC against neighboring constructs and lay out its constructivist foundation. A fifth section presents the propositional model and its boundary conditions. The article closes with implications for design, work, and education, as well as a research agenda.

## 2 The homogenization debate and its missing level of analysis

The vocabulary now used to discuss generative AI and sameness was built to describe collectives, and the confusion it produces when applied to individuals is a confusion of levels. Algorithmic monoculture is a property of a market or an ecology of decision-makers. It holds when many actors lean on a common predictive signal, and the dangers it names, such as correlated failure and depressed collective welfare, are dangers of the aggregate rather than experiences of any single actor within it (Kleinberg and Raghavan, 2021). Outcome homogenization is similarly relational. It is defined by the tendency of shared components, whether training data or models, to produce systematically similar results across many people (Zhang et al., 2025). Model collapse refers to a generative process rather than a person, namely the way a model’s output distribution narrows when the model is trained on material previously produced by earlier models (Shumailov et al., 2024). The reduction in collective diversity reported by Doshi and Hauser (2024) is, by construction, a statistic computed over a set of texts; in their study, the individual texts grew more creative even as the set grew less varied.

Each of these is a statement about something other than a mind, and the slide from such statements to claims about human creativity is exactly what a person-level construct has to interrupt. A market can be monocultural, yet the people within it feel distinctive. A distribution of outputs can narrow while a particular author feels original. The reverse also holds: a person can feel boxed in by sameness, even as the objective range of what she could obtain remains wide. Two short cases make the point. A writer who prompts a model in her habitual way may receive a run of structurally similar drafts and conclude that the system produces only one kind of thing; her PTC is high even though a differently worded request would have returned ample variety. A second writer, surrounded by genuinely varied output but attending only to its surface polish, may never register the variation; her PTC is low even though the objective diversity in front of her is high. The two appraisals come apart from the aggregate, and from each other, which is the basic reason a separate construct is needed.

The same decoupling appears at the level of a group, where its practical stakes are clearer. Consider a product team in which every member draws on the same model to generate concepts. The objective diversity of what the model can return may be substantial. Still, if the members converge on similar prompts and read the results through a shared expectation of sameness, the team may collectively conclude that the design space has been exhausted and stop searching. Here, a high average level of PTC, formed from a narrow and shared sample of the model’s output, produces a real behavioral effect, namely premature closure, that no measure of the model’s objective range would predict. The lesson is not that objective homogenization is irrelevant, but that it reaches behavior only through perception, and that perception can run ahead of or behind the facts of the option space.

The appraisal also separates from objective homogenization for a more general reason. Appraisals are formed from limited and often unrepresentative samples, and people are not reliably sensitive to the quality of the evidence on which their impressions rest. Whether PTC tracks any objective index of homogenization is therefore an empirical question rather than something the definition guarantees. This is not a weakness peculiar to PTC. It is the ordinary situation of subjective constructs, and it is what makes the appraisal worth studying in its own right: if perception drove behavior only when it matched the aggregate, there would be little reason to measure perception at all.

A second body of recent work approaches the person directly but stops short of a construct that organizes the findings. Studies of human-AI interaction report that people who write with AI often do not feel that the resulting text is theirs, even when they put their names to it, a pattern named the ghostwriter effect (Draxler et al., 2024). Surveys of knowledge workers find that greater confidence in AI is associated with less critical engagement, and that the perceived effort of careful thinking declines as reliance grows (Gerlich, 2025). Broader evidence links frequent reliance on AI tools to greater cognitive offloading and weaker performance on tests of independent reasoning (Gerlich, 2025), and a recurring observation is that the fluency of an AI-produced result is easily mistaken for understanding that the user does not, in fact, possess. These studies describe consequences, namely lost ownership, reduced effort, and weaker reasoning. They do not name the appraisal that lies upstream of those consequences, nor do they explain why the same conditions might produce both confidence and disengagement. PTC is offered as that upstream appraisal, and the convergence paradox as the reason its effects diverge.

The proliferation of terms is itself informative. Within a few years, the literature has produced monoculture, outcome homogenization, model collapse, and the loss of collective diversity, along with looser coinages in commentary, each foregrounding a different facet of one broad worry. Such proliferation usually marks a field that has identified a phenomenon faster than it has agreed on what the phenomenon is. It is the kind of situation in which conceptual work earns its keep(Attwood, 2022). The contribution of the present analysis is not to add another aggregate-level label, but to supply the missing person-level construct. Once that level is in place, the aggregate terms can be read as describing the conditions under which PTC is more or less likely to form, and PTC can be read as the channel through which those conditions reach behavior. The two levels are complementary rather than competing, and keeping them distinct is what prevents claims about output distributions from being mistaken for claims about psychology.

## 3 Defining perceived template convergence

A construct earns its place by being defined precisely enough to be told apart from its neighbors. We define perceived template convergence as a graded appraisal, held by an individual, that the construals she can obtain through generative AI are collapsing toward a common template. Three features of this definition deserve comment. The appraisal is person-level, so it is a property of a construing subject rather than of a market or a corpus. It is graded rather than all-or-nothing, so it admits of degree and can rise or fall with experience. And it concerns the relation among a set of possible outputs, the sense that they are tending toward one form, rather than the qualities of any single output considered alone. The object of the appraisal is the construal, by which we mean a way of framing or rendering a task: an angle on a problem, a structure for a document, a treatment of an image. PTC is the judgment that the menu of such construals on offer is narrowing.

### 3.1 Perceived template convergence and processing fluency

The nearest psychological neighbor is processing fluency, the subjective ease with which information is taken in (Reber et al., 2004; Alter and Oppenheimer, 2009; Oppenheimer, 2008; Schwarz et al., 2021). Fluency and PTC are both metacognitive feelings, and they will often be correlated, since templated, convergent material tends to be easy to process. They are nonetheless distinct in two ways that matter for the model developed below. First, fluency attaches to the experience of a single stimulus, whereas PTC attaches to a perceived relation across many stimuli. A person can take in a stimulus easily while judging it to be unlike anything else she has seen, and she can labor over a stimulus she regards as entirely typical. Second, the two feelings differ in tone. Fluency is generally pleasant and is read as a cue to truth, familiarity, or safety, which is part of why it promotes sharing and imitation (Reber et al., 2004). PTC is mixed in tone, carrying at once the comfort of a result that feels reliably attainable and the unease of a result that feels like everyone else’s. The model turns on that mixed tone, and treating PTC as if it were fluency would obscure the very feature that makes it consequential.

### 3.2 Perceived template convergence and attitudes toward algorithms

A second neighbor is the set of attitudes studied under the concepts of algorithm appreciation and algorithm aversion. People sometimes favor algorithmic over human judgment (Logg et al., 2019) and sometimes turn away from an algorithm after seeing it err (Dietvorst et al., 2015, 2018), with the balance shifting according to the task and to whether it is seen as objective or subjective (Castelo et al., 2019; Longoni et al., 2019). These are stances toward using algorithmic advice. PTC is not a stance toward use but an appraisal of content, concerning whether the advice, once obtained, is perceived to be converging. The two can vary independently. A person who appreciates algorithms may perceive little convergence, and a person who perceives strong convergence may continue to rely on the tool, in part because convergence makes its output feel safe. Distinguishing appraisal of content from disposition toward use is necessary if the analysis is to ask how appraisals of convergence, rather than general orientations toward technology, shape what people go on to do.

### 3.3 What the construct does and does not claim

Stating what PTC is not helps fix what it is, since a new construct is defined as much by its boundaries as by its content. PTC is not a claim that convergence is occurring; that is the aggregate question addressed by the homogenization literature, and a person high in PTC may be mistaken. PTC is not a competence or a skill, although competence will turn out to moderate its effects. It is not an attitude toward AI in general, and it is not the same as trust, which concerns reliance on the tool rather than the perceived sameness of what the tool returns. Nor is PTC a perception of low quality; a person may judge AI output to be excellent and convergent at the same time, and the model below treats high perceived quality and high convergence as the combination most likely to raise tool-bound confidence. Marking these boundaries does more than tidy the conceptual space. It identifies the discriminant-validity tests that a PTC measure must pass, against fluency and quality judgments on one side and against trust and general AI attitudes on the other, and specifies the conditions under which PTC should and should not predict the outcomes the model assigns to it.

Because PTC is graded and formed from experience, it should also be dynamic. Early encounters with a tool may produce little sense of convergence, since the user has not yet seen enough output to judge the relation among possible results. As use accumulates, and as the user notices the resemblance among what the tool returns and among what peers produce with similar tools, the appraisal can strengthen. It can also weaken, for instance when a user finds that varied prompting yields varied construals, or when contact with work made without AI restores a sense of how wide the option space can be. This dynamic character matters for the research agenda, since it implies that PTC is a moving quantity best captured over time rather than a fixed trait, and it foreshadows the self-reinforcing loop that the model describes. Table 1 places PTC alongside its aggregate- and individual-level neighbors, providing for each its level of analysis, what it describes, and the feature that distinguishes it from PTC.

**Table 1:** Conceptual disambiguation of perceived template convergence (PTC) from adjacent aggregate-level and individual-level constructs.

Construct	Level of analysis	What it describes	Representative source	Distinction from PTC
Algorithmic monoculture	System/market	Many decision-makers relying on a common predictive signal	Kleinberg and Raghavan (2021)	An aggregate property of a decision ecosystem, not a person’s perception
Outcome homogenization	Population	Shared models or data producing similar outcomes across persons	Zhang et al. (2025)	Relational and population-level; PTC is one person’s appraisal
Model collapse	Model/data process	Narrowing of a model’s output distribution under recursive training	Shumailov et al. (2024)	A property of the generative process, external to the user’s mind
Collective-diversity loss	Output set	Reduced variety across a set of human and AI outputs	Doshi and Hauser (2024)	A statistic over outputs; PTC is the perception, which can diverge from it
Processing fluency	Individual	Subjective ease of processing a single stimulus	Reber et al. (2004); Schwarz et al. (2021)	Concerns one stimulus and is positive in tone; PTC concerns the relation among many outputs and is mixed in tone
Algorithm appreciation/aversion	Individual	Attitude toward using algorithmic advice	Logg et al. (2019); Dietvorst et al. (2015)	An attitude toward use, not an appraisal of whether the content is converging
Perceived template convergence	Individual	Appraisal that AI-supplied construals are collapsing toward a common template	This article	Focal construct of this analysis

## 4 A constructivist account of perceived template convergence

A construct defined by perception and meaning calls for a theory of how people make meaning, and personal construct theory provides one. The account in this section proceeds in four steps. It first sets out the view of the person as a construer. It then interprets AI templates as construals supplied from outside that person. It next reads reliance on convergent templates through the theory of proxy agency. It finally connects the resulting changes to the experience of authorship and meaning, thereby preparing the model’s two-channel structure.

### 4.1 The person as construer

Kelly’s (1955) personal construct theory begins from the premise that people understand the world by building and testing systems of bipolar personal constructs, and that, in the theory’s fundamental postulate, a person’s processes are channelized by how she anticipates events (Kelly, 1955). The recurring image is of the person as a kind of scientist, someone who does not passively absorb reality but actively construes it, who selects among alternative constructions, and who revises them as her anticipations are borne out or disappointed. Kelly named this stance constructive alternativism, the view that any event can be construed in more than one way and that the person is free, within limits, to choose among construals (Walker and Winter, 2007). Several of the theory’s corollaries bear on the present problem. The individuality corollary holds that people differ in how they construe the same event. The experience corollary holds that a construct system is elaborated as the person construes the succession of events she meets. The choice corollary holds that a person tends to adopt the pole of a construct through which she anticipates the fuller development of her system. The sociality corollary holds that to take part in a social process is, in part, to construe how others construe. Together these commitments make construing an active, effortful, and personal achievement., and they place the elaboration of one’s own construct system at the center of psychological functioning.

These corollaries are not antiquarian detail; they specify what is at stake when construing is handed to a machine. The choice corollary, for instance, holds that a person moves toward whichever construction promises to extend her system, which is to say that healthy construing is oriented toward growth rather than mere coverage. A construal adopted wholesale from a tool may cover the task at hand while extending nothing, since it has not been built from, or tested against, the person’s own anticipations. The experience corollary makes the same point over time: a construct system develops only as its owner construes a succession of events for herself. Hence, a long run of imported, unexamined construals leaves the system static even as output accumulates. And the sociality corollary implies that when many people construe through the same tool, each may come to anticipate the others as converging too, which is one route by which an individual appraisal of convergence can be socially amplified.

### 4.2 Templates as outsourced construals

Read through this lens, a generative AI template is a construal that originates outside the person. A template offers a ready way to frame a question, organize a document, or render an image, and to take it up is to import a construction rather than to build one. Imported construals are, in themselves, neither new nor troubling. Construing has always drawn on culture, genre, and example, and people routinely make sense of unfamiliar tools through shared interpretive frames (Orlikowski and Gash, 1994). What is distinctive about generative AI is the scale and immediacy with which finished construals are supplied, the authority they carry as the apparent output of a capable system (Epley et al., 2007), and the prospect that, across many users drawing on the same models, the imported construals are not merely borrowed but shared. PTC is the appraisal that this sharing has gone as far as collapse, that the menu of construals has narrowed toward one. In Kelly’s terms, a high level of PTC registers a contraction in the range of alternatives through which the person might anticipate. The pressure it creates runs counter to the elaboration of her own construct system and toward what can be called foreclosure, a settling into imported construals that are no longer tested against her own constructions. The cost of foreclosure, on this account, is not that the imported construal is wrong. It is that construing has migrated out of the person, and with it the activity that the theory places at the heart of psychological life.

A brief example fixes the idea. A designer asked to produce a brand identity might once have generated the defining contrasts herself, weighing, say, playful against austere or traditional against modern, and arriving at a position she could defend as her own. Prompted instead for a finished identity, she receives a polished construal in which the system has already resolved those contrasts, and resolved, she suspects, in much the way they are resolved for everyone who prompts similarly. If she treats the result as a draft to interrogate, her construing is engaged, and the import is harmless. If she treats it as the answer, the contrasts that would have organized her own construct system are never drawn, and the activity the theory regards as central is quietly skipped. Foreclosure, in this sense, is less a dramatic loss than an omission that is easy not to notice.

### 4.3 Proxy agency and personal efficacy

The migration of construing has an agentic side that social cognitive theory states directly. Bandura (2001, 2006) distinguishes three modes through which people exercise agency. They act individually, by their own efforts; they act through proxies, by getting others, and we would add tools, to secure outcomes on their behalf; and they act collectively, by pooling effort. Agency in this account works through intentionality, forethought, self-reaction, and self-reflection (Bandura, 1989, 2006). Reliance on generative AI is a clear case of proxy agency, since the person obtains a result through the system’s capabilities rather than through the exercise and development of her own. Bandura is deliberately two-sided about proxy agency. It can be efficient and sensible, sparing effort that is better spent elsewhere. It carries, though, a specific hazard: a person who secures outcomes without exercising her own competencies may leave those competencies undeveloped, and her sense of personal efficacy, as distinct from confidence in the proxy, may weaken (Bandura, 1997, 2006). This article argues that PTC is the appraisal that turns this standing hazard into an operating dynamic. When the proxy’s outputs are perceived as varied, the person still has reason to bring her own construing to bear, if only to choose among options. When they are perceived as converging, the outputs come to appear reliably substitutable for her own construing, and the conditions under which proxy reliance erodes personal agency are met.

The distinction between confidence in the proxy and a person’s own efficacy is worth drawing sharply, because the model below turns on it. A programmer who relies on a code-generating assistant may become highly confident that working code will appear, and that confidence is well founded as long as the assistant performs. It is nonetheless confidence in a capability she accesses rather than one she holds, and it can rise even as her own command of the underlying material stays flat or declines. The healthy case Bandura describes is reciprocal, in that efficacy beliefs both reflect and build genuine competence through mastery experience (Bandura, 1997). Proxy reliance breaks the loop, since the mastery experience accrues, in effect, to the tool. Perceived convergence sharpens the break by removing the residual reason to engage one’s own competence, namely the need to choose among genuinely different options. So, it is the condition under which proxy confidence and personal efficacy are most likely to part ways.

### 4.4 Authorship, ownership, and meaning

The final element is meaning. The experience of work as meaningful, as significant and worth doing, is among the steadiest correlates of motivation, engagement, and well-being (Rosso et al., 2010; Hackman and Oldham, 1976; Deci and Ryan, 2000; Ryan and Deci, 2000; Gagné and Deci, 2005; Spreitzer, 1995; Steger et al., 2012). One durable source of meaning is authorship, the sense that a piece of work expresses one’s own agency and is, in a real psychological sense, one’s own. Research on psychological ownership locates that sense in the investment of the self in a target, in control over it, and in close knowledge of it (Pierce et al., 2001, 2003; Van Dyne and Pierce, 2004), and research on job crafting shows that people reshape their work to make it more fully an expression of who they are (Wrzesniewski and Dutton, 2001; Rudolph et al., 2017). Generative AI bears directly on this source of meaning. Experiments find that producing text with AI lowers felt ownership even when the person formally claims authorship, the ghostwriter effect, and that ownership returns only as the person’s own investment in the work rises (Draxler et al., 2024). When construals are supplied by a proxy and, beyond that, perceived as shared with everyone using similar tools, the bases of felt ownership weaken together, as self-investment, control, and distinctiveness all decline. So too do the experiences of autonomy and competence that self-determination theory identifies as nutriments of intrinsic motivation (Ryan and Deci, 2000). The convergence the person perceives is thus not only an agentic matter but a matter of meaning, and the two combine to produce the model’s central tension.

Authorship under shared tools has a relational edge that deserves note. Part of what makes a piece of work feel like one’s own is that it is recognizably not someone else’s, and distinctiveness is among the bases of psychological ownership for that reason (Pierce et al., 2003). When the construals a person works from are perceived as shared by everyone using similar tools, distinctiveness is threatened not only at the level of the individual output but also at the level of the self that the output is meant to express. The worry is no longer simply that a given document is generic; it is that one’s manner of working, and the product that manner yields, are interchangeable with anyone else’s. This is one reason PTC should bear more heavily on meaning than a private perception of low quality would. A result can be poor, yet idiosyncratically one’s own, and a result can be excellent yet, if perceived as convergent, fail to register as an expression of the person at all.

Pulling the four steps together, the account implies that PTC should reach behavior along two channels whose effects oppose each other. Through an agentic channel, the perception that competent output is reliably attainable by proxy should raise a particular, tool-bound kind of confidence. Through an authorship channel, the perception that the available construals are shared rather than one’s own should reduce experienced authorship and meaning. The next section turns these two channels into a model. It specifies their consequences for two different intentions: to keep producing within the prevailing template and to depart from it.

## 5 The convergence paradox: a propositional model

The account just given can be assembled into a model, shown in Figure 1, that places PTC at its center. In the model, an antecedent pattern of behavior, reliance on generative AI, gives rise to the appraisal; the appraisal feeds two mediating mechanisms with opposing signs; and those mechanisms feed two contrasting intentions. Two boundary conditions govern the links most likely to vary across people. Because the analysis is conceptual, the relationships in Figure 1 are propositions about the expected sign and direction of each path rather than estimates from data, and no coefficients are reported. We emphasize at the outset, and not only in the limitations, that none of P1 through P6 or M1 and M2 has yet been empirically tested: the model’s present warrant is the plausibility of the theories from which it is assembled rather than any direct evidence for the paradox. The findings that motivate it document homogenization rather than its perception, and they now span the aggregate level (Doshi and Hauser, 2024), the individual and collective diversity of creative output (Moon et al., 2025), and individual ideation, where users of a language model produced more and more detailed ideas yet felt less responsible for them (Anderson et al., 2024), a pattern that echoes the paradox while stopping short of testing it. None of these studies measure perceived template convergence as such, or show that PTC varies across individuals or predicts what the model claims. We present the propositions in the order in which the model builds them, and we compile them with their rationale in Table 2.

**Figure 1:**
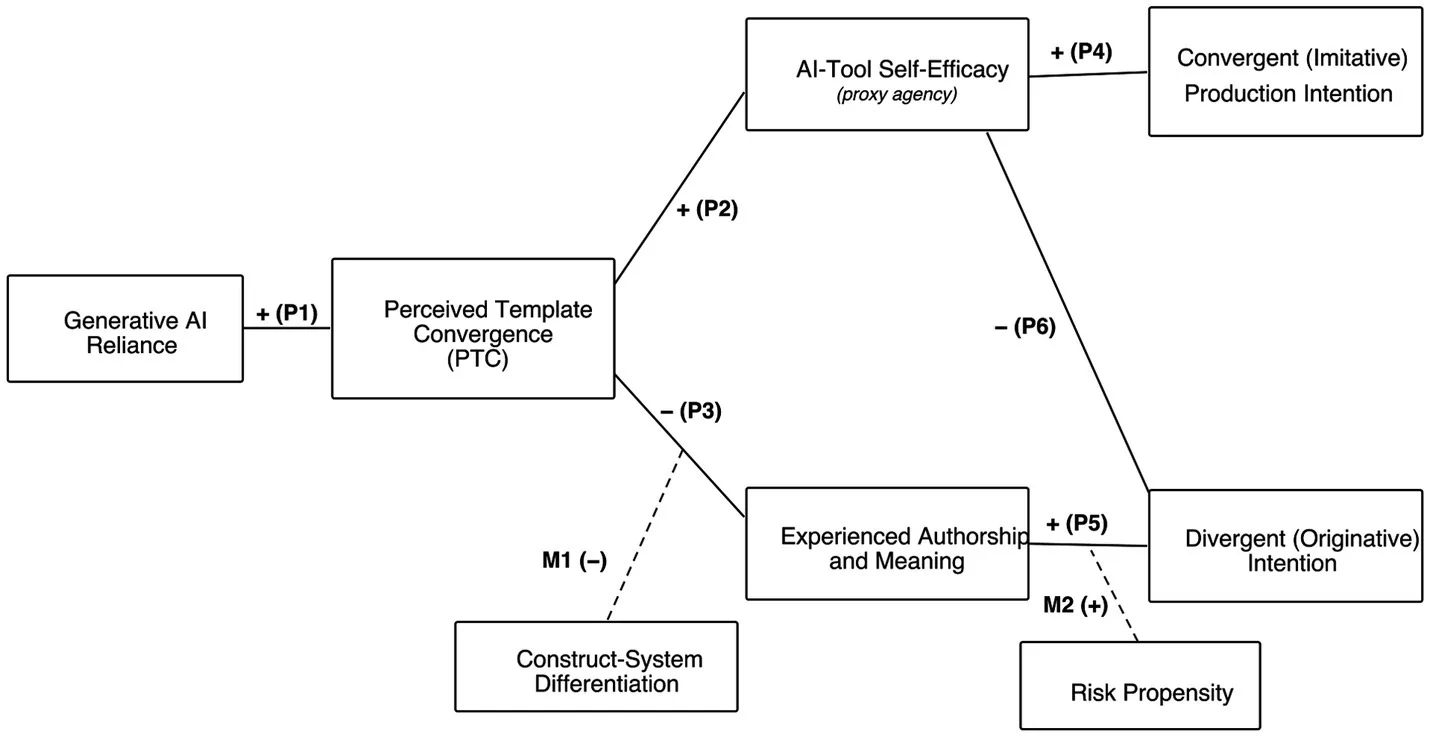
The convergence-paradox model. Boxes are constructs and arrows are theoretical propositions. Solid arrows represent the directional propositions P1–P6; dashed arrows represent the moderating propositions M1 and M2. A plus or minus sign on an arrow indicates the proposed sign of that relationship. The model is conceptual; the arrows denote proposed relationships and no path coefficients are estimated.

**Table 2:** Propositions of the convergence-paradox model, with proposed sign and theoretical rationale.

Path	Proposition	Proposed sign	Theoretical rationale (key sources)
P1	Generative AI reliance on PTC	+	Greater delegation to common models increases exposure to convergent output and occasions to notice the resemblance (Kleinberg and Raghavan, 2021; Salganik et al., 2006)
P2	PTC to AI-tool self-efficacy	+	Convergence makes competent output feel predictable and attainable by proxy; fluency is read as competence (Bandura, 2001; Fisher et al., 2015; Compeau and Higgins, 1995)
P3	PTC to experienced authorship and meaning	−	Pre-fabricated, shared construals undercut self-investment, control, and distinctiveness; construct-system foreclosure (Kelly, 1955; Draxler et al., 2024; Pierce et al., 2003; Rosso et al., 2010)
P4	AI-tool self-efficacy to convergent production intention	+	Proxy confidence in working with convergent output; expressed as fast, low-risk reproduction (Bandura, 1997; Doshi and Hauser, 2024)
P5	Experienced authorship and meaning to originative intention	+	Felt authorship and meaning supply the intrinsic motivation that sustains effortful origination (Deci and Ryan, 2000; Wrzesniewski and Dutton, 2001; Krueger et al., 2000)
P6	AI-tool self-efficacy to originative intention	−	Good-enough satisficing under proxy agency crowds out costly construing; offloading reduces independent engagement (Bandura, 2006; Sparrow et al., 2011; Risko and Gilbert, 2016; Gerlich, 2025)
M1	Construct-system differentiation moderating PTC to authorship	Buffers (−)	Differentiated construers re-construe templates as material, preserving authorship (Kelly, 1955; Walker and Winter, 2007)
M2	Risk propensity moderating authorship to originative intention	Amplifies (+)	Risk tolerance strengthens conversion of preserved meaning into intention to depart from the template (Krueger et al., 2000; Ajzen, 1991)

The model enters with the link from reliance to appraisal. As a person delegates more of her construing to generative systems, using them more often, on more consequential tasks, and with less revision afterward, she accumulates more exposure to the output of a small number of common models and more occasion to notice the resemblance among results. The structural fact that many users draw on shared models (Kleinberg and Raghavan, 2021) combines with a well-documented tendency for shared inputs to pull choices toward common ground, observed for example in cultural markets where information about others’ selections reduces the variety of what people go on to choose (Salganik et al., 2006; Muthukrishna and Henrich, 2016). Together, these give the appraisal of convergence ample material on which to form. This yields Proposition P1.

*Proposition P1*. Greater reliance on generative AI is positively associated with perceived template convergence.

The appraisal then sends two signals at once, and their simultaneity is the source of the paradox. The first signal concerns competence. We use AI-tool self-efficacy for a person’s belief in her capacity to produce competent output through the AI, a proxy and tool-bound relative of the technology self-efficacy long studied in information systems (Compeau and Higgins, 1995; Davis, 1989; Venkatesh et al., 2003). Perceived convergence should raise it. When the construals a tool returns are seen as settling toward a dependable template, a serviceable result comes to feel predictable and within reach, whatever the task, and the fluency of that result is readily taken as the user’s own capability (Bandura, 2001; Fisher et al., 2015). The second signal concerns authorship. We use experienced authorship and meaning for the felt sense that a piece of work expresses one’s own construing and is significant and one’s own (Rosso et al., 2010; Pierce et al., 2003). Perceived convergence should lower it. If the construals on offer are supplied by a proxy and, further, seem shared with everyone using similar tools, then self-investment, control, and distinctiveness are all undercut, as the ghostwriter effect illustrates for AI-assisted text (Draxler et al., 2024). In Kelly’s terms, the person is anticipating through imported rather than self-built constructions, which is foreclosure of her construct system (Kelly, 1955). The two signals give Propositions P2 and P3.

*Proposition P2*. Perceived template convergence is positively associated with AI-tool self-efficacy.

*Proposition P3*. Perceived template convergence is negatively associated with experienced authorship and meaning.

The two mechanisms feed two intentions that differ in their relation to the prevailing template. Convergent production intention is the intention to keep producing within that template, reproducing the dependable form the tool supplies. Originative intention is the intention to depart from the template toward work that is recognizably one’s own, which is the motivational core of creative and entrepreneurial action (Amabile, 1983; Runco and Jaeger, 2012; Sternberg and Lubart, 1996; Krueger et al., 2000; Liñán and Chen, 2009). The mechanisms map onto these intentions as their natures suggest. Tool-bound confidence is confidence in getting good results by working with convergent output, and its natural expression is the quick, low-risk reproduction through which competent mutually similar work accumulates (Doshi and Hauser, 2024; Bandura, 1997). Experienced authorship and meaning, by contrast, supply the intrinsic motivation that sustains the slower and less certain project of making something that leaves the common form behind (Deci and Ryan, 2000; Wrzesniewski and Dutton, 2001). Hence, Propositions P4 and P5 are presented.

*Proposition P4*. AI-tool self-efficacy is positively associated with convergent production intention.

*Proposition P5*. Experienced authorship and meaning are positively associated with originative intention.

Were the model to stop here, it would describe two parallel channels and nothing more. The paradox arises from a further link that connects them: the confidence that perceived convergence confers should also depress originative intention. The mechanism is satisficing under proxy agency. When a competent result is reliably on hand from the template, the additional effort of leaving it, of doing the slower and riskier construing that origination requires, comes to look unnecessary, and the proxy’s adequate output crowds out the development of the person’s own. This is the hazard Bandura attaches to proxy reliance, the leaving undeveloped of competencies one does not exercise (Bandura, 1997, 2006), and it is consistent in spirit with evidence that habitual cognitive offloading accompanies reduced independent engagement (Sparrow et al., 2011; Risko and Gilbert, 2016; Fisher et al., 2015; Storm and Stone, 2015; Gerlich, 2025). That evidence, however, concerns the offloading of memory and reasoning rather than creative origination, so carrying it over to originative intention is a theoretical extrapolation rather than a documented finding, and we flag Proposition P6 as the most tentative link in the model, offered for testing rather than assumed. With this link, the paradox is complete: perceived convergence strengthens the route to imitation and, through the same confidence, weakens the route to origination.

*Proposition P6*. AI-tool self-efficacy is negatively associated with originative intention.

Taken together, the six propositions describe a tendency for perceived convergence to sustain itself, which we call the convergence trap. Reliance feeds the appraisal; the appraisal raises tool-bound confidence and lowers authorship; confidence drives further on-template production while suppressing origination; and the loss of authorship removes the main motivational support for leaving the template. The model therefore predicts not a simple effect of convergence on creativity but a split in behavior, in which an appraisal that feels empowering steers effort away from exactly the original work whose growing scarcity first raised concern. Table 2 sets out the propositions with their rationale and principal sources.

### 5.1 Boundary conditions

The trap is a tendency rather than a certainty, and two boundary conditions govern when it tightens. The first is internal to the person and follows from the constructivist foundation. Construct systems differ in their differentiation, the number and independence of the constructs a person brings to a domain, a property studied as cognitive complexity within personal construct theory and beyond it (Kelly, 1955; Walker and Winter, 2007). A person with a well-differentiated system can re-construe an imported template as raw material rather than as a finished answer, seeing in a convergent output one option among several that her own system can develop. For her, perceived convergence need not erode authorship, because construing remains hers to do. For a person with a sparse system, the template arrives as the answer, with little internal structure to set against it, and the meaning-eroding effect of convergence operates without check. We therefore expect differentiation to buffer the negative path from convergence to authorship.

*Proposition M1*. Construct-system differentiation weakens the negative relationship between perceived template convergence and experienced authorship and meaning.

The second boundary condition concerns the step from authorship to action. Even where authorship and meaning are preserved, leaving a prevailing template is a risky move, since it gives up a dependable result for an uncertain one, and people differ in their tolerance for such risk. Risk propensity is an established antecedent of entrepreneurial and creative intention (Krueger et al., 2000; Ajzen, 1991; Zhao et al., 2005), and it should govern how readily preserved meaning translates into the actual intention to originate. Among the risk-tolerant, meaning converts readily into the intention to depart from the template; among the risk-averse, the same meaning is more likely to remain inert, held in check by the safety of the convergent option.

*Proposition M2*. Risk propensity strengthens the positive relationship between experienced authorship and meaning and originative intention.

### 5.2 The model at work

The two cases that follow are illustrative: they trace the model’s internal logic and are not evidence for it. Consider first an experienced essayist who uses a language model daily. She is heavily reliant, and by Proposition P1 her perceived convergence is high, since she has seen enough of the model’s output to feel its family resemblance keenly. By Proposition P2, her confidence in producing competent drafts through the tool is correspondingly high. Yet her construct system for prose is richly differentiated, so by Proposition M1 the path from convergence to lost authorship is buffered: she reads each draft as one option among many that her own system can rework, and her sense of authorship survives. Her differentiation and her tolerance for the risk of departing from a serviceable draft mean that preserved meaning converts into originative intention, as Propositions P5 and M2 describe, and the crowd-out pressure of Proposition P6, though present, does not prevail. For her, convergence is a manageable feature of a useful tool rather than a trap.

Consider now a novice in the same domain. He, too, is heavily reliant and high in perceived convergence, and his tool-bound confidence is, if anything, higher, since he has little independent standard against which to check the fluency of the model’s output. His construct system is sparse, so Proposition M1 offers little buffering, and the path from convergence to lost authorship operates at nearly full strength. With authorship low, the motivational route to origination through Proposition P5 is weak to begin with, and his risk aversion further dampens whatever remains. The satisficing pressure of Proposition P6 meets little resistance. He produces fluent, on-template work, feels capable while doing so, and has neither the construing resources nor the motivation to depart from the form the tool supplies. For him, unlike for the essayist, the same conditions close the trap described by the model. The contrast is the point: PTC does not act alone but through a configuration of construct-system differentiation and risk propensity that determines whether perceived convergence liberates or confines.

## 6 Discussion

The contribution of the analysis is, first of all, conceptual. By naming perceived template convergence and situating it at the level of individual appraisal, it provides the person-level construct that the homogenization literature has lacked and separates claims about markets, output distributions, and data-generating processes from claims about minds. Monoculture, outcome homogenization, and the loss of collective diversity describe aggregates (Kleinberg and Raghavan, 2021; Doshi and Hauser, 2024; Zhang et al., 2025); PTC describes how a person registers and responds to such an aggregate condition, in a way that need not match it. The move recasts the question of algorithmic homogenization as one about human construing, namely whether, and under what conditions, people keep authoring their own constructions when finished ones are supplied in convergent form. The account fits evidence that the best human originators still outperform current AI on open-ended divergent thinking (Koivisto and Grassini, 2023), and it connects to long-standing findings that creativity requires both novelty and usefulness and is shaped by the constraints under which it unfolds (Runco and Jaeger, 2012; Acar et al., 2019; Anderson et al., 2014).

The analysis also draws together three lines of theory that are rarely combined. It reads AI templates through Kelly’s (1955) constructivism as outsourced construals, interprets reliance on them through Bandura’s (2001, 2006) account of proxy agency, with that account’s warning about the wasting of unused competence, and ties the resulting loss to the place of authorship and ownership in meaningful work (Rosso et al., 2010; Pierce et al., 2003; Draxler et al., 2024). The convergence paradox is what this combination yields: a single appraisal that raises confidence and lowers authorship at once, thereby dividing behavior. The paradox, in turn, connects to the debate over whether AI augments or automates human work, which holds that the same technology can extend or replace human capabilities depending on how it is used (Raisch and Krakowski, 2021). PTC offers a psychological reading of when augmentation tips into substitution for the individual, namely when convergence is perceived, confidence is gained by proxy, and authorship is given up.

The practical implications turn on the boundary conditions rather than on convergence as such. For the designers of generative systems, the analysis suggests that preserving perceived divergence matters as much as preserving objective diversity. Interfaces that present several visibly different construals, keep the user’s own contribution in view, and avoid returning a single authoritative answer may blunt both the inflation of confidence and the loss of authorship, in keeping with evidence that greater self-investment restores ownership of AI-assisted work (Draxler et al., 2024). A complementary and more direct lever is to diversify the system’s outputs themselves: recent experimental work finds that prompting a model to take on several distinct personas can preserve, rather than flatten, the diversity of collaboratively generated ideas (Wan and Kalman, 2026), a constructive counterpoint to the aggregate homogenization reported by Doshi and Hauser (2024) and a concrete means of lowering perceived convergence at its source. For managers and educators who care about original work, the lever is authorship. Protecting the conditions of felt ownership, namely self-investment, control, and distinctiveness, should keep open the motivational route to origination even under heavy AI use. In contrast, arrangements that reduce a person to checking convergent output should be expected to close it. For education in particular, two emphases follow. One is AI literacy, as a way of keeping the appraisal accurate and the confidence honest (Ng et al., 2021), so that learners neither overstate convergence nor mistake templated fluency for understanding, a corrective to the link among AI confidence, offloading, and weakened reasoning (Gerlich, 2025; Fisher et al., 2015). The other, less obvious, is the deliberate building of learners’ own construct systems, since it is differentiation that lets a template be received as material rather than as an answer. A teaching practice that cultivates several independent ways of construing a subject is, on this account, a direct defense against the trap.

Because the construct is new, the priority for research is measurement. PTC calls for an instrument that captures the perceived narrowing of the option space and demonstrates discriminant validity against objective indices of homogenization on one side and against processing fluency, quality judgments, trust, and general AI attitudes on the other (Reber et al., 2004; Logg et al., 2019). With such an instrument, the propositions become testable. The mediating roles of tool-bound confidence and authorship, the crossing path that constitutes the paradox, and the buffering and amplifying roles of differentiation and risk propensity can each be examined, and the model’s opposing indirect effects suit designs that can separate them. Because the analysis insists that perceived and objective convergence can come apart, the two should be separated by design rather than conflated. Objective template diversity can be held constant while perceived convergence is manipulated through framing or prompt structure, and, conversely, objective diversity can be varied while the manner of presentation is held fixed, so that a factorial crossing of objective diversity with perceived convergence isolates the effect the model attributes to perception rather than to the state of the option space. Objective diversity, measured directly, should also enter tests of P1 through P6 as a covariate rather than being left implicit in the antecedent of reliance. Following users over time would let the trap, which is dynamic by nature, be observed as it tightens or loosens.

A further moderator, which we deliberately leave outside the core model and outside Figure 1, concerns the accuracy of the appraisal itself. AI literacy, the competencies needed to understand, evaluate, and work critically with AI systems (Ng et al., 2021; Carolus et al., 2023), should shape both how readily PTC forms and how strongly it inflates tool-bound confidence, since a more literate user can recognize that apparent convergence may be an artifact of how she is prompting and can discount the fluency of a templated result as a sign of her own skill. AI literacy might therefore weaken the path from reliance to PTC and the path from PTC to confidence, loosening the trap. We flag it as a promising but as yet unplaced third moderator whose position in the model, unlike the specified roles of construct-system differentiation and risk propensity, is a question for future theoretical and empirical work rather than a claim we make here.

Several limits bound the analysis. It is a conceptual analysis and reports no data, so its propositions are reasoned rather than shown, and their fate is open. The signs it proposes are theory-based and may prove to depend on context; the path from confidence to origination, in particular, may vary with how origination is operationalized and with the domain. The constructs need operational measures and tests of discriminant validity before the model can be assessed, and the boundary conditions named here are unlikely to exhaust the relevant ones. The account is, as presented, culturally unmarked, yet construing is culturally situated, and the meaning of authorship, the tone attached to convergence, and the readiness to leave a shared form may all differ across cultural settings in ways that touch every path in the model. Finally, the analysis treats PTC as a property of individuals; convergence can also be construed collectively, and a group-level counterpart, namely how teams and communities appraise and respond to shared templates, remains to be developed.

## 7 Conclusion

Generative AI has made it ordinary to think with construals that are not one’s own, and easy to mistake their convergence for one’s own competence. The concern that this is flattening human creativity has so far been voiced in the language of systems and outputs. The argument here is that the decisive variable lies a level down, in the person: not whether outputs are in fact converging, but whether a person perceives that they are, and what that perception does to her sense of authorship and her intention to originate. Perceived template convergence names that variable, and the convergence paradox states its two-sidedness, the way a single appraisal can raise confidence and erode authorship together. Whether the age of generative AI narrows human construing or merely furnishes it will depend, in part, on conditions that the analysis has sought to specify: the literacy with which people read their tools, the richness of the construct systems they bring to them, and the degree to which the experience of authorship is preserved. None of these is fixed, which is the hopeful side of the account. The trap the model describes is a tendency, and the same boundary conditions that allow it to close are the levers by which it can be held open. Naming the construct is a first step toward measuring it, and measuring it is a first step toward keeping the option space, and the construing that fills it, genuinely open.
